# Trivalent inactivated influenza vaccine effective against influenza A(H3N2) variant viruses in children during the 2014/15 season, Japan

**DOI:** 10.2807/1560-7917.ES.2016.21.42.30377

**Published:** 2016-10-20

**Authors:** Norio Sugaya, Masayoshi Shinjoh, Chiharu Kawakami, Yoshio Yamaguchi, Makoto Yoshida, Hiroaki Baba, Mayumi Ishikawa, Mio Kono, Shinichiro Sekiguchi, Takahisa Kimiya, Keiko Mitamura, Motoko Fujino, Osamu Komiyama, Naoko Yoshida, Kenichiro Tsunematsu, Atsushi Narabayashi, Yuji Nakata, Akihiro Sato, Nobuhiko Taguchi, Hisayo Fujita, Machiko Toki, Michiko Myokai, Ichiro Ookawara, Takao Takahashi

**Affiliations:** 1 Keiyu Hospital, Yokohama, Kanagawa, Japan; 2 Keio University School of Medicine, Tokyo, Japan; 3 Yokohama City Institute of Public Health, Yokohama, Kanagawa, Japan; 4 National Hospital Organization, Tochigi Medical Center, Utsunomiya, Tochigi, Japan; 5 Sano Kousei General Hospital, Sano, Tochigi, Japan; 6 Fuji Heavy Industries Health Insurance Society Ota Memorial Hospital, Ota, Gunma, Japan; 7 Saitama City Hospital, Saitama, Saitama, Japan; 8 National Hospital Organization Saitama National Hospital, Wako, Saitama, Japan; 9 Tokyo Metropolitan Ohtsuka Hospital, Tokyo, Japan; 10 Eiju General Hospital, Tokyo, Japan; 11 Saiseikai Central Hospital, Tokyo, Japan; 12 National Hospital Organization, Tokyo Medical Center, Tokyo, Japan; 13 Kyosai Tachikawa Hospital, Tachikawa, Tokyo, Japan; 14 Hino Municipal Hospital, Hino, Tokyo, Japan; 15 Kawasaki Municipal Hospital, Kawasaki, Kanagawa, Japan; 16 Nippon Kokan Hospital, Kawasaki, Kanagawa, Japan; 17 Yokohama Municipal Citizen's hospital, Yokohama, Kanagawa, Japan; 18 Hiratsuka Kyosai Hospital, Hiratsuka, Kanagawa, Japan; 19 Hiratsuka City Hospital, Hiratsuka, Kanagawa, Japan; 20 Shizuoka City Shimizu Hospital, Shizuoka, Shizuoka, Japan; 21 Japanese Red Cross Shizuoka Hospital, Shizuoka, Shizuoka, Japan

**Keywords:** Influenza, Influenza Virus, Laboratory surveillance, vaccine and immunization, vaccine effectiveness

## Abstract

The 2014/15 influenza season in Japan was characterised by predominant influenza A(H3N2) activity; 99% of influenza A viruses detected were A(H3N2). Subclade 3C.2a viruses were the major epidemic A(H3N2) viruses, and were genetically distinct from A/New York/39/2012(H3N2) of 2014/15 vaccine strain in Japan, which was classified as clade 3C.1. We assessed vaccine effectiveness (VE) of inactivated influenza vaccine (IIV) in children aged 6 months to 15 years by test-negative case–control design based on influenza rapid diagnostic test. Between November 2014 and March 2015, a total of 3,752 children were enrolled: 1,633 tested positive for influenza A and 42 for influenza B, and 2,077 tested negative. Adjusted VE was 38% (95% confidence intervals (CI): 28 to 46) against influenza virus infection overall, 37% (95% CI: 27 to 45) against influenza A, and 47% (95% CI: -2 to 73) against influenza B. However, IIV was not statistically significantly effective against influenza A in infants aged 6 to 11 months or adolescents aged 13 to 15 years. VE in preventing hospitalisation for influenza A infection was 55% (95% CI: 42 to 64). Trivalent IIV that included A/New York/39/2012(H3N2) was effective against drifted influenza A(H3N2) virus, although vaccine mismatch resulted in low VE.

## Introduction

Influenza vaccination is the most effective method of preventing influenza virus infection and its potentially severe complications. Based on the results of randomised controlled trials [1,2] and observational studies [3,4] the vaccine effectiveness (VE) of inactivated influenza vaccine (IIV) in healthy children has been reported to be 40% to 70%.

During the 2014/15 season, a variant strain of influenza A(H3N2) virus that was classified as phylogenetic clade 3C.2a and was genetically distinct from the 2014/15 A/Texas/50/2012(H3N2)-like clade 3C.1 vaccine reference strain appeared in the northern hemisphere. Consistent with the substantial vaccine mismatch, no or low VE against A(H3N2) was reported as interim estimates in Canada, the United Kingdom (UK), and the United States (US) [5-7].

There have been many reports of VE in studies conducted by a test-negative case–control (TNCC) design. Most of the subjects of the studies were adults and the elderly, and VE in children was not fully elucidated, especially the VE of IIV in children. In 2014, it was clearly recommended in the US that live attenuated influenza vaccine (LAIV) be used in healthy children from 2 to 8 years of age [8]. However, the effectiveness of LAIV against influenza A(H1N1)pdm09 in the 2013/14 season was found to be poor [9,10]. Moreover, although one large randomised trial reported superior relative efficacy of LAIV over IIV against antigenically drifted influenza A(H3N2) viruses [11], neither LAIV nor IIV provided significant protection against the drifted influenza A(H3N2) viruses in children in the 2014/15 season, and LAIV did not provide greater protection than IIV against these viruses [8]. Accordingly, LAIV is no longer recommended over IIV in children aged 2–8 years in the US [12].

In the past, Japan's strategy for controlling influenza was to vaccinate schoolchildren, based on the theory that this could reduce influenza epidemics in the community, and a special programme to vaccinate schoolchildren against influenza was begun in 1962. However, the programme was discontinued in 1994 because of lack of evidence that it had limited the spread of influenza in the community [13]. At present in Japan, influenza vaccination is officially recommended for elderly and high-risk patients with underlying conditions. However, ca 50% of children receive an influenza vaccination every year on their parents’ initiative, paid for out of pocket [14].

Only trivalent IIV was approved for use in children in Japan until the 2014/15 season, and we have previously reported on the VE of IIV in children in Japan based on the results of influenza rapid diagnostic tests (IRDT) during the 2013/14 season [14], when influenza A(H1N1)pdm09 and B viruses were the main epidemic strains. VE was high against influenza A (63%, 95% CI: 56 to 69), and especially high (77%, 95% CI: 59 to 87) against influenza A(H1N1)pdm09, but was only 26% against influenza B (95% CI: 14 to 36).

A large influenza epidemic caused by A(H3N2) occurred in the 2014/15 season, and that provided an excellent opportunity to test VE against A(H3N2) virus infection in children. Influenza A(H3N2) outbreaks were reported throughout Japan since week 44 of 2014. The epidemic peaked between week 51 of 2014 and the week 1 of 2015. The start and peak of the influenza epidemic in the 2014/15 season occurred 3 weeks earlier than in the average year [15]. The vaccine strain used in Japan for influenza A(H3N2) was A/New York/39/2012(H3N2), which is different from A/Texas/50/2012; however, it belongs to the same clade, 3C.1.

We investigated the VE of trivalent IIV in children during the large epidemic caused by the drifted influenza A(H3N2) virus by conducting a study by using the TNCC design and based on IRDT results.

## Methods

### Epidemiology

According to FluNet [16], 5,070 influenza A(H3N2) viruses were detected in Japan from week 45 of 2014 to week 14 of 2015, but only 50 A(H1N1) pdm09 viruses and 598 influenza B viruses were detected during the same period. In the 2014/15 season, over 99% of the influenza A viruses detected were A(H3N2) viruses (5,070/5,120).

### Phylogenetic analysis

Influenza A(H3N2) viruses were isolated by using MDCK or MDCK-AX4 cells at the Yokohama City Institute of Public Health, Yokohama, Kanagawa, Japan [17]. The nucleotide sequences of the haemagglutinin (HA) genes were subjected to phylogenetic analysis, and phylogenetic trees were constructed using MEGA 6 software (The Biodesign Institute, Arizona, USA) and the neighbour-joining method [18]. The viruses were isolated in the 2014/15 influenza seasons. The nucleotide sequences determined are available from the Global Initiative on Sharing All Influenza Data (GISAID) EpiFlu database. Accession numbers for the HA genes are EPI679784-EPI679834, respectively (Table 1).

**Table 1:** Details of the influenza A(H3N2) haemagglutinin sequences obtained from the Global Initiative on Sharing All Influenza Data (GISAID)’s EpiFlu database used in the phylogenetic analysis for this study.

Segment ID	Isolate name	Collection date	Country	Originating laboratory	Submitting laboratory	Authors
EPI679784	A/YOKOHAMA/30/2014	27/1/2014	Japan	Yokohama City Institute of Public Health	Yokohama City Institute of Public Health	Kawakami C, Usuku S, Sasao T, Mizuno T
EPI679785	A/YOKOHAMA/56/2014	29/2/2014	Japan	Yokohama City Institute of Public Health	Yokohama City Institute of Public Health	Kawakami C, Usuku S, Sasao T, Mizuno T
EPI679786	A/YOKOHAMA/82/2014	9/3/2014	Japan	Yokohama City Institute of Public Health	Yokohama City Institute of Public Health	Kawakami C, Usuku S, Sasao T, Mizuno T
EPI679787	A/YOKOHAMA/88/2014	13/4/2014	Japan	Yokohama City Institute of Public Health	Yokohama City Institute of Public Health	Kawakami C, Usuku S, Sasao T, Mizuno T
EPI679788	A/YOKOHAMA/168/2014	12/12/2014	Japan	Yokohama City Institute of Public Health	Yokohama City Institute of Public Health	Kawakami C, Usuku S, Sasao T, Mizuno T
EPI679789	A/YOKOHAMA/100/2014	5/11/2014	Japan	Yokohama City Institute of Public Health	Yokohama City Institute of Public Health	Kawakami C, Usuku S, Sasao T, Mizuno T
EPI679790	A/YOKOHAMA/101/2014	15/11/2014	Japan	Yokohama City Institute of Public Health	Yokohama City Institute of Public Health	Kawakami C, Usuku S, Sasao T, Mizuno T
EPI679791	A/YOKOHAMA/104/2014	18/11/2014	Japan	Yokohama City Institute of Public Health	Yokohama City Institute of Public Health	Kawakami C, Usuku S, Sasao T, Mizuno T
EPI679792	A/YOKOHAMA/109/2014	25/11/2014	Japan	Yokohama City Institute of Public Health	Yokohama City Institute of Public Health	Kawakami C, Usuku S, Sasao T, Mizuno T
EPI679793	A/YOKOHAMA/113/2014	25/11/2014	Japan	Yokohama City Institute of Public Health	Yokohama City Institute of Public Health	Kawakami C, Usuku S, Sasao T, Mizuno T
EPI679794	A/YOKOHAMA/134/2014	1/12/2014	Japan	Yokohama City Institute of Public Health	Yokohama City Institute of Public Health	Kawakami C, Usuku S, Sasao T, Mizuno T
EPI679773 /EPI679795	A/YOKOHAMA/138/2014	2/12/2014	Japan	Yokohama City Institute of Public Health	Yokohama City Institute of Public Health	Kawakami C, Usuku S, Sasao T, Mizuno T
EPI679774 /EPI679796	A/YOKOHAMA/14/2015	13/1/2015	Japan	Yokohama City Institute of Public Health	Yokohama City Institute of Public Health	Kawakami C, Usuku S, Sasao T, Mizuno T
EPI679797	A/YOKOHAMA/150/2014	1/12/2014	Japan	Yokohama City Institute of Public Health	Yokohama City Institute of Public Health	Kawakami C, Usuku S, Sasao T, Mizuno T
EPI679798 /EPI679775	A/YOKOHAMA/154/2014	5/12/2014	Japan	Yokohama City Institute of Public Health	Yokohama City Institute of Public Health	Kawakami C, Usuku S, Sasao T, Mizuno T
EPI679799 /EPI679776	A/YOKOHAMA/159/2014	4/12/2014	Japan	Yokohama City Institute of Public Health	Yokohama City Institute of Public Health	Kawakami C, Usuku S, Sasao T, Mizuno T
EPI679800 /EPI679777	A/YOKOHAMA/16/2015	13/1/2015	Japan	Yokohama City Institute of Public Health	Yokohama City Institute of Public Health	Kawakami C, Usuku S, Sasao T, Mizuno T
EPI679801	A/YOKOHAMA/176/2014	15/12/2014	Japan	Yokohama City Institute of Public Health	Yokohama City Institute of Public Health	Kawakami C, Usuku S, Sasao T, Mizuno T
EPI679802	A/YOKOHAMA/182/2014	23/12/2014	Japan	Yokohama City Institute of Public Health	Yokohama City Institute of Public Health	Kawakami C, Usuku S, Sasao T, Mizuno T
EPI679803	A/YOKOHAMA/183/2014	20/12/2014	Japan	Yokohama City Institute of Public Health	Yokohama City Institute of Public Health	Kawakami C, Usuku S, Sasao T, Mizuno T
EPI679804	A/YOKOHAMA/184/2014	25/12/2014	Japan	Yokohama City Institute of Public Health	Yokohama City Institute of Public Health	Kawakami C, Usuku S, Sasao T, Mizuno T
EPI679805	A/YOKOHAMA/30/2015	16/1/2015	Japan	Yokohama City Institute of Public Health	Yokohama City Institute of Public Health	Kawakami C, Usuku S, Sasao T, Mizuno T
EPI679806	A/YOKOHAMA/42/2015	23/1/2015	Japan	Yokohama City Institute of Public Health	Yokohama City Institute of Public Health	Kawakami C, Usuku S, Sasao T, Mizuno T
EPI679807	A/YOKOHAMA/48/2015	29/1/2015	Japan	Yokohama City Institute of Public Health	Yokohama City Institute of Public Health	Kawakami C, Usuku S, Sasao T, Mizuno T
EPI679808	A/YOKOHAMA/5/2015	6/1/2015	Japan	Yokohama City Institute of Public Health	Yokohama City Institute of Public Health	Kawakami C, Usuku S, Sasao T, Mizuno T
EPI679809 /EPI679778	A/YOKOHAMA/58/2015	26/1/2015	Japan	Yokohama City Institute of Public Health	Yokohama City Institute of Public Health	Kawakami C, Usuku S, Sasao T, Mizuno T
EPI679810 /EPI679779	A/YOKOHAMA/60/2015	4/2/2015	Japan	Yokohama City Institute of Public Health	Yokohama City Institute of Public Health	Kawakami C, Usuku S, Sasao T, Mizuno T
EPI679811	A/YOKOHAMA/65/2015	6/2/2015	Japan	Yokohama City Institute of Public Health	Yokohama City Institute of Public Health	Kawakami C, Usuku S, Sasao T, Mizuno T
EPI679812 /EPI679780	A/YOKOHAMA/68/2015	6/2/2015	Japan	Yokohama City Institute of Public Health	Yokohama City Institute of Public Health	Kawakami C, Usuku S, Sasao T, Mizuno T
EPI679813	A/YOKOHAMA/72/2015	16/2/2015	Japan	Yokohama City Institute of Public Health	Yokohama City Institute of Public Health	Kawakami C, Usuku S, Sasao T, Mizuno T
EPI679814	A/YOKOHAMA/74/2015	13/2/2015	Japan	Yokohama City Institute of Public Health	Yokohama City Institute of Public Health	Kawakami C, Usuku S, Sasao T, Mizuno T
EPI679815	A/YOKOHAMA/8/2015	10/1/2015	Japan	Yokohama City Institute of Public Health	Yokohama City Institute of Public Health	Kawakami C, Usuku S, Sasao T, Mizuno T
EPI679816	A/YOKOHAMA/84/2015	14/3/2015	Japan	Yokohama City Institute of Public Health	Yokohama City Institute of Public Health	Kawakami C, Usuku S, Sasao T, Mizuno T
EPI679781 /EPI679817	A/YOKOHAMA/85/2015	19/3/2015	Japan	Yokohama City Institute of Public Health	Yokohama City Institute of Public Health	Kawakami C, Usuku S, Sasao T, Mizuno T
EPI679818	A/YOKOHAMA/86/2015	20/3/2015	Japan	Yokohama City Institute of Public Health	Yokohama City Institute of Public Health	Kawakami C, Usuku S, Sasao T, Mizuno T
EPI679819	A/YOKOHAMA/87/2015	27/3/2015	Japan	Yokohama City Institute of Public Health	Yokohama City Institute of Public Health	Kawakami C, Usuku S, Sasao T, Mizuno T
EPI679820	A/YOKOHAMA/88/2015	18/4/2015	Japan	Yokohama City Institute of Public Health	Yokohama City Institute of Public Health	Kawakami C, Usuku S, Sasao T, Mizuno T
EPI679821	A/YOKOHAMA/97/2014	13/11/2014	Japan	Yokohama City Institute of Public Health	Yokohama City Institute of Public Health	Kawakami C, Usuku S, Sasao T, Mizuno T
EPI679822	A/YOKOHAMA/98/2014	17/11/2014	Japan	Yokohama City Institute of Public Health	Yokohama City Institute of Public Health	Kawakami C, Usuku S, Sasao T, Mizuno T
EPI679823	A/YOKOHAMA/149/2014	6/12/2014	Japan	Yokohama City Institute of Public Health	Yokohama City Institute of Public Health	Kawakami C, Usuku S, Sasao T, Mizuno T
EPI679824	A/YOKOHAMA/156/2014	5/12/2014	Japan	Yokohama City Institute of Public Health	Yokohama City Institute of Public Health	Kawakami C, Usuku S, Sasao T, Mizuno T
EPI679825	A/YOKOHAMA/171/2014	12/12/2014	Japan	Yokohama City Institute of Public Health	Yokohama City Institute of Public Health	Kawakami C, Usuku S, Sasao T, Mizuno T
EPI679826	A/YOKOHAMA/2/2015	5/1/2015	Japan	Yokohama City Institute of Public Health	Yokohama City Institute of Public Health	Kawakami C, Usuku S, Sasao T, Mizuno T
EPI679827	A/YOKOHAMA/33/2015	16/1/2015	Japan	Yokohama City Institute of Public Health	Yokohama City Institute of Public Health	Kawakami C, Usuku S, Sasao T, Mizuno T
EPI679828	A/YOKOHAMA/89/2014	27/9/2014	Japan	Yokohama City Institute of Public Health	Yokohama City Institute of Public Health	Kawakami C, Usuku S, Sasao T, Mizuno T
EPI679829	A/YOKOHAMA/91/2014	20/10/2014	Japan	Yokohama City Institute of Public Health	Yokohama City Institute of Public Health	Kawakami C, Usuku S, Sasao T, Mizuno T
EPI679830 /EPI679782	A/SHINJYUKU/1/2014	30/12/2014	Japan	Yokohama City Institute of Public Health	Yokohama City Institute of Public Health	Kawakami C, Usuku S, Sasao T, Mizuno T
EPI679831 /EPI679783	A/SETAGAYA/3/2014	20/12/2014	Japan	Yokohama City Institute of Public Health	Yokohama City Institute of Public Health	Kawakami C, Usuku S, Sasao T, Mizuno T
EPI679832	A/ZAMA/1/2015	20/3/2015	Japan	Yokohama City Institute of Public Health	Yokohama City Institute of Public Health	Kawakami C, Usuku S, Sasao T, Mizuno T
EPI679833	A/ZAMA/2/2014	20/11/2014	Japan	Yokohama City Institute of Public Health	Yokohama City Institute of Public Health	Kawakami C, Usuku S, Sasao T, Mizuno T
EPI679834	A/ISEHARA/1/2014	17/11/2014	Japan	Yokohama City Institute of Public Health	Yokohama City Institute of Public Health	Kawakami C, Usuku S, Sasao T, Mizuno T

### Study enrolment and location

Children aged 6 months to 15 years with a fever of 38 °C or over and cough and/or rhinorrhoea and who had received an IRDT in an outpatient clinic of one of 20 hospitals between 10 November 2014 and 31 March 2015 were enrolled in this study. In Japan, the cost of IRDT is covered by public health insurance, and almost all children with a high fever of 38 °C or over receive an IRDT during an influenza epidemic. Our hospitals were located in six (Gunma, Tochigi, Saitama, Tokyo, Kanagawa, and Shizuoka prefectures) of the 47 prefectures in Japan, mainly in the Greater Tokyo Metropolitan area.

Patients who met the symptom criteria were eligible if they had not received antiviral medication before enrolment. Patients who had been vaccinated against influenza less than 14 days before illness onset were excluded from this study. A TNCC design was used to estimate VE based on IRDT results as previously described [14].

### Diagnosis of influenza

Nasopharyngeal swabs were obtained from all of the enrollees. Several different IRDT kits, including the Espline Influenza A and B-N kit (Fujirebio Inc., Tokyo, Japan), ImmunoAce FLU kit with LineJudge pdm kit (Tauns Laboratories, INC, Shizuoka, Japan), Quick Chaser Flu A, B kit (Mizuho Medy Co., Ltd., Saga, Japan), and QuickNavi-Flu kit (DENKA SEIKEN Co., Ltd., Tokyo, Japan), all of which are capable of differentiating between influenza A and influenza B, were used in the hospitals. Two of the 20 participating hospitals used the LineJudge pdm kit, which enables differentiation between influenza A, influenza B, and influenza A(H1N1)pdm09. According to their respective manuals, all of the IRDT kits used in this study have similar sensitivities (88‒100%) and specificities (94‒100%) [19].

### Case and control patient identification

The IRDT-positive patients were enrolled as case patients and the IRDT-negative patients as control patients. Their medical charts were reviewed, and information regarding symptoms, influenza vaccination, number of vaccine doses (one or two), influenza complications and hospitalisations, sex, age, comorbidities, and treatment with neuraminidase inhibitors (NAIs) was collected and recorded. Children were excluded if definite information on influenza vaccination was found to be unavailable.

When a child was brought to one of our clinics, the parents or guardians were asked about the child’s influenza vaccination status; the status was then usually confirmed by consulting the Maternal and Child Health Handbook provided by local governments, in which all vaccinations are recorded by the doctors in charge.

### Vaccine

A trivalent inactivated subunit-antigen vaccine was used to vaccinate children in Japan during the 2014/15 season. The vaccine strains used to produce the vaccine for use in the 2014/15 season were: A/California/7/2009(X-179A) for protection against A(H1N1)pdm09, A/New York/39/2012(X-233A) for protection against A(H3N2), and B/Massachusetts/02/2012(BX-51B) for protection against type B, Yamagata lineage.

In Japan, two 0.25 ml doses of vaccine 2 to 4 weeks apart are recommended for children aged 6 months to 2 years, and two 0.5 ml doses of vaccine 2 to 4 weeks apart are recommended for children aged 3‒12 years. Only one 0.5 ml dose of vaccine is recommended for children aged 13 years and over.

### Test-negative case–control design

We estimated VE by TNCC design. VE was defined as 1 - OR (odds ratio), and was calculated as described below.

### Statistical analysis

Statistical analysis was performed by using SPSS 22.0 software (IBM, US) and Ekuseru-Toukei 2015 for Windows software programme (Social Survey Research Information Co., Ltd., Tokyo, Japan).

VE was adjusted for age group (6–11 months, 1‒2 years, 3‒5 years, 6‒12 years, and 13‒15 years), comorbidity (yes or no), area of the Kanto region of Japan, i.e. north area: Gunma Prefecture and Tochigi Prefecture; middle area: Saitama Prefecture and Tokyo Prefecture; and south area: Kanagawa Prefecture and Shizuoka Prefecture, and month of illness onset.

The influenza season was divided into an early phase (November, December and January) and a late phase (February and March), and the VE for each phase was compared. We also estimated VE according to the number of doses of vaccine administered. The Breslow-Day test was used to assess the homogeneity of the odds ratios in several 2 x 2 contingency tables. P value of < 0.05 was considered to indicate statistical significance.

### VE against hospitalisation

We calculated the VE against hospitalisation using the TNCC design. The cases included patients with positive IRDT results who were admitted to hospital. These cases were divided into an in-patient group that had received the influenza vaccine and a in-patient group that had not received a vaccine. The control group included all patients who were not admitted to hospital, whether they received an influenza vaccine or not. Admitted patients with negative IRDT results were excluded from the analysis.

### Ethics

This study was approved by the Keio University Ethics Committee in 2013 (Approval Number 20130216) and by the Institutional Review Board (IRB) at each hospital. Eligible patients and their guardians (usually parents) were verbally informed of the objective and methods of the study in the outpatient departments. The requirement for obtaining written consent was waived by the IRBs because performing an IRDT is standard practice in Japan.

## Results

### Influenza A(H3N2) virus characterisation

**Figure 1:**
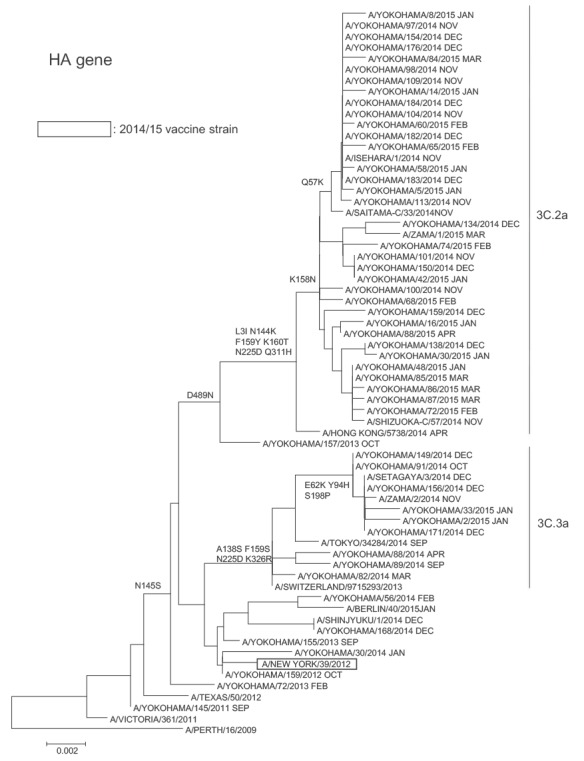
Phylogenetic analysis with sequences of the HA1 subunit of the haemagglutinin gene from reference viruses and influenza A(H3N2) sequences derived from children aged 6 months to 15 years, Yokohama, Japan, November 2014 to March 2015

The HA sequences of the majority of the 128 influenza A(H3N2) viruses in the 2014/15 season that were sequenced (113/128; 88.3%) were further characterised within this clade as belonging to subclade 3C.2a of clade 3C.2, with fewer (15/128; 11.7%) belonging to clade 3C.3 (Figure 1). These subclade 3C.2a viruses are considered genetically distinct from both the A/New York/39/2012 (H3N2) clade 3C.1 vaccine strain used in Japan and the A/Texas/50/2012 WHO vaccine reference strain.

The amino acids are described with the H1 numbering. The amino acid substitutions relative to the A/PERTH/16/2009 virus are shown to the left of the nodes.

Reference viruses downloaded from the Global Initiative on Sharing All Influenza Data (GISAID) EpiFlu database (EPI679784-EPI679835).

### Characteristics of the enrollees

A total of 3,896 children were enrolled in this study, of whom 144 were subsequently excluded from the analysis for the following reasons: 117 were < 6 months old or > 15 years old, or their age was unknown; two had a fever < 38 °C; 24 had an unclear influenza vaccination history and the date of one patient’s clinic visit had not been recorded. 

**Figure 2:**
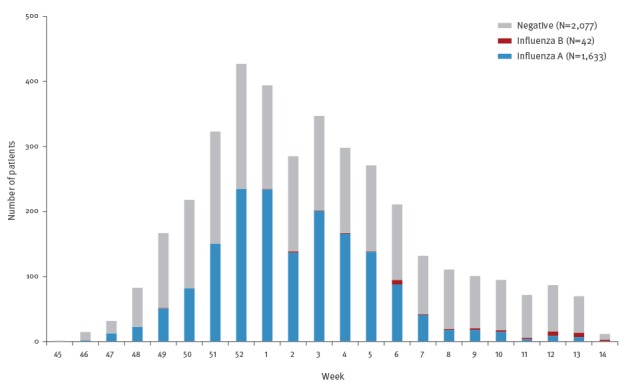
Influenza patients aged 6 months to 15 years diagnosed with influenza rapid diagnostic tests by week and type of virus in influenza vaccine effectiveness evaluation, Japan, November 2014 to March 2015 (n=3,752)

Of the remaining 3,752 patients who were eligible for inclusion in the analysis in this study, 1,633 had influenza A (1 had influenza A(H1N1)pdm09 infection, and the remaining 1,632 had influenza A, subtype unknown); and 42 patients had influenza B. Of the 3,752 patients included, 2,077 were IRDT-negative. Figure 2 shows the total numbers of cases of influenza diagnosed by week at the 20 hospitals as a whole. The first case of influenza A was diagnosed in week 45 of 2014. The number of influenza A cases diagnosed per week increased towards the end of 2014, and peaked in week 52, after which time the number of cases diagnosed per week gradually decreased. A small number of influenza B cases were seen after week 6 of 2015.

Table 2 shows the characteristics of the enrollees. The comorbidities consisted of respiratory comorbidities (n = 403), neurological comorbidities (n = 98), cardiac comorbidities (n = 41), allergic comorbidities (n = 38), renal comorbidities (n = 21), endocrinological comorbidities (n = 22), immunological comorbidities (n = 14), and other comorbidities (n = 88).

**Table 2:** Characteristics of the children aged 6 months to 15 years enrolled in influenza vaccine effectiveness study, Japan, November 2014 to March 2015 (n = 3,752)

Characteristic	Any influenza (%)		Influenza A (%)	Influenza B (%)	Influenza negative (%)	Difference between ‘any influenza’ and ‘influenza negative’
Sex	Female	799 (48)	777 (48)	22 (52)	965 (46)	p = 0.4575^a^
	Male	876 (52)	856 (52)	20 (48)	1,111 (54)	
	**Total**	**1,675**	**1,633**	**42**	**2,076**	
Age	6–11 mo	47 (3)	44 (3)	3 (7)	136 (7)	p < 0.001^b^
	1–2 y	229 (14)	224 (14)	5 (12)	738 (36)	
	3–5 y	410 (24)	402 (25)	8 (19)	574 (28)	
	6–12 y	793 (47)	772 (47)	21 (50)	519 (25)	
	13–15 y	196 (12)	191 (12)	5 (12)	110 (5)	
	**Total**	**1,675**	**1,633**	**42**	**2,077**	
Comorbidity	No	1,343 (82)	1,307 (82)	36 (86)	1,585 (79)	p = 0.0251^a^
	Yes	293 (18)	287 (18)	6 (14)	418 (21)	
	**Total**	**1,636**	**1,594**	**42**	**2,003**	
Area of Kanto region^c^	North	125 (7)	121 (7)	4 (10)	170 (8)	p = 0.4007^d^
	Middle	781 (47)	766 (47)	15 (36)	996 (48)	
	South	769 (46)	746 (46)	23 (55)	911 (44)	
	**Total**	**1,675**	**1,633**	**42**	**2,077**	
Month of illness onset	Nov 2014	38 (2)	38 (2)	0 (0)	93 (4)	p < 0.001^e^
	Dec 2014	646 (39)	644 (39)	2 (5)	699 (34)	
	Jan 2015	742 (44)	737 (45)	5 (12)	614 (30)	
	Feb 2015	188 (11)	175 (11)	13 (31)	385 (19)	
	Mar 2015	61 (4)	39 (2)	22 (52)	286 (14)	
	**Total**	**1,645**	**1,633**	**42**	**2,077**	
Clinic visit (hours after symptom onset)	< 12 h	551 (34)	541 (34)	10 (24)	602 (31)	p = 0.0348^f^
	12–48 h	994 (61)	968 (61)	26 (63)	1,114 (57)	
	> 48 h	80 (5)	75 (5)	5 (12)	251 (13)	
	**Total**	**1,625**	**1,584**	**41**	**1,967**	
	> 12 h	1,074	1,043	31	1,365	
Received vaccine in 2014/15 season	No	978 (58)	952 (58)	26 (62)	930 (45)	p < 0.001^a^
	Yes	697 (42)	681 (42)	16 (38)	1,147 (55)	
	**Total**	**1,675**	**1,633**	**42**	**2,077**	
Vaccine doses received in 2014/15 season	None	978 (59)	952 (58)	26 (63)	930 (45)	p < 0.001^g^
	One	224 (13)	220 (14)	4 (10)	336 (16)	
	Two	464 (28)	457 (28)	11 (27)	807 (39)	
	**Total**	**1,670**	**1,629**	**41**	**2,073**	
Treatment with neuraminidase inhibitors	No	45 (4)	44 (4)	1 (3)	1,409 (98)	p < 0.001^h^
	Yes	1,231 (96)	1,201 (96)	30 (97)	29 (2)	
	**Total**	**1,276**	**1,245**	**31**	**1,438**	

^a^ Chi-squared test.

^b^ Chi-squared test, Cramer's V = 0.3188.

^c^ Area of Kanto region. North: Gunma Prefecture and Tochigi Prefecture; Middle: Saitama Prefecture and Tokyo Prefecture; South: Kanagawa Prefecture and Shizuoka Prefecture.

^d^ Chi-squared test, Cramer's V = 0.0221.

^e^ Chi-squared test, Cramer's V = 0.2367.

^f^ Chi-squared test, comparing the number of patients who came to the clinic < 12 hours after the onset with the number who came later.

^g^ Chi-squared test, Cramer's V = 0.1379.

^h^ Chi-squared test, Cramer's V = 0.9453.

Of the children with positive IRDT, 95.1% (1,545/1,625) had been brought to the hospital or clinic and diagnosed within 48 hours of illness onset, and 96.5% (1,231/1,276) of the children with a positive IRDT were treated with NAIs (Table 2).

### Vaccine effectiveness against influenza

The adjusted VE of the influenza vaccine was 38% (95% CI: 28 to 46) against influenza virus infection overall (Table 3), 37% (95% CI: 27 to 45) against influenza A infection, and 47% (95% CI: -2 to 73) against influenza B infection (Table 3).

**Table 3:** Effectiveness of trivalent inactivated influenza vaccine, influenza vaccine effectiveness study, Japan, November 2014 to March 2015 (n = 3,752)

Category		Any influenza^a^		Influenza A^a^		Influenza B ^a,b^	
		VE% (95% CI)	Vaccinated/cases (Vaccinated/controls)	VE% (95% CI)	Vaccinated/cases (Vaccinated/controls)	VE% (95% CI)	Vaccinated/cases (Vaccinated/controls)
All ages 6 months to 15 years	Crude	42 (34 to 49)	697/1,675 (1,147/2,077)	42 (34 to 49)	681/1,633 (1,147/2,077)	50 (6 to 73)	16/42 (1,147/2,077)
	Adjusted ^c, d^	38 (28 to 46)		37 (27 to 45)		47 (-2 to 73)	
	Adjusted ^c,d,e^	39 (30 to 47)		39 (29 to 47)		51 (4 to 75)	
	Adjusted ^c,d,f^	39 (27 to 49)		38 (26 to 48)		65 (21 to 85)	
Age 6–11 months	Crude	-8 (-137 to 51)	11/47 (30/136)	-18 (-161 to 47)	11/44 (30/136)	NA	
	Adjusted ^c^	3 (-119 to 57)		-5 (-139 to 54)			
Age 1–2 years	Crude	42 (21 to 57)	106/229 (440/738)	40 (19 to 56)	105/224 (440/738)		
	Adjusted ^c^	41 (20 to 57)		40 (18 to 56)			
Age 3–5 years	Crude	54 (41 to 65)	181/410 (364/574)	55 (42 to 65)	176/402 (364/574)		
	Adjusted ^c^	54 (40 to 65)		55 (41 to 65)			
Age 6–12 years	Crude	29 (11 to 43)	336/793 (264/519)	29 (11 to 43)	327/772 (264/519)		
	Adjusted ^c^	26 (7 to 41)		25 (6 to 41)			
Age 13–15 years	Crude	41 (5 to 64)	63/196 (49/110)	40 (3 to 63)	62/191 (49/110)		
	Adjusted ^c^	41 (1 to 65)		41 (0 to 65)			

NA: not analysed.

^a^ One hospital had no information on comorbidity.

^b^ Not analysed by age because few patients developed influenza.

^c^ Adjusted for comorbidity (yes or no), area (north area, middle area, south of the Kanto region), month of onset.

^d^ Adjusted for age (0–15 years).

^e^ Adjusted for time tested after the onset (< 12, 12–48 and > 48 hours).

^f^ Only patients tested > 12 hours after onset.

VE by age group was analysed only in regard to influenza A. Statistically significant adjusted VE was not demonstrated in the infant group aged 6 months to 11 months, in which it was -5% (95% CI: -139 to 54), but statistically significant adjusted VE was seen in the 1- to 12-year-old group. Moderate adjusted VE against influenza A was demonstrated in the 1- to 2-year-old group (40%, 95% CI: 18 to 56) and in the 3- to 5-year-old group (55%, 95% CI: 41 to 65). Adjusted VE against influenza A in the 6- to 12-year-old group was lower (25%, 95% CI: 6 to 41), and it was not statistically significant in the 13- to 15-year-old group (41%, 95% CI: -0.1 to 65). Crude VE against influenza A was 29% (95% CI: 11 to 43) in the 6- to 12-year-old group and was significantly lower than the 55% (95% CI: 42 to 65) in the 3- to 5-year-old group (p = 0.0089, Breslow-Day test).

VE against influenza B was not analysed by age group because of the small number of cases.

### Protection against hospitalisation

Patients admitted to the hospitals with influenza A were divided into an unvaccinated group (n = 231) and a vaccinated group (n = 104) (Table 4). The control group consisted of patients who were not admitted to the hospital, including 1,447 unvaccinated patients and 1,439 vaccinated patients. Influenza vaccination was effective in preventing hospitalisation for influenza A virus infection (55%, 95% CI: 42 to 64) (Table 4), but VE was not statistically significant in preventing hospitalisation for influenza B virus infection because of the small number of cases.

**Table 4:** Effectiveness of trivalent inactivated influenza vaccine in preventing influenza hospitalisation, influenza vaccine effectiveness study, Japan, November 2014 to March 2015 (n=3,228)

Influenza type	Vaccination status	No hospitalisation	Hospitalisation for influenza	Effectiveness in preventing influenza hospitalisation	95% CI
Any Influenza	Unvaccinated	1,447	236	55	43 to 64
	Vaccinated	1,439	106		
Type A	Unvaccinated	1,447	231	55	42 to 64
	Vaccinated	1,439	104		
Type B	Unvaccinated	1,447	5	60	-108 to 92
	Vaccinated	1,439	2		

CI: confidence interval.

Admitted patients with negative IRDT results (n = 143) were excluded from this analysis.

### Vaccine effectiveness by month of illness onset

Crude VE against influenza A infection decreased markedly in the late phase of the influenza epidemic, from 46% (95% CI: 37 to 54) in the 3-month period November, December, and January to 13% (95% CI: -18 to 36) in the 2-month period February and March (Table 5).

**Table 5:** Effectiveness of trivalent inactivated influenza vaccine, by phase of the influenza season, influenza vaccine effectiveness study, Japan, November 2014 to March 2015 (n=3,752)

Phase of the influenza season	Any influenza		Influenza A		Influenza B	
	VE% (95%CI)	Vaccinated/cases (Vaccinated/controls)	VE% (95% CI)	Vaccinated/cases (Vaccinated/controls)	VE% (95% CI)	Vaccinated/cases (Vaccinated/controls)
Nov 2014 –Jan 2015	46 (38 to 54)	573/1,426 (781/1,406)	46 (37 to 53)	572/1,419 (781/1,406)	87 (-11 to 98)	1/7 (781/1,406)
Feb–Mar 2015	17 (-11 to 38)	124/249 (366/671)	13 (-18 to 36)	109/214 (366/671)	38 (-24 to 69)	15/35 (366/671)
**Total**	**42 ** **(34 to 49)**	**697/1,675 (1,147/2,077)**	**42 ** **(34 to 49)**	**681/1,633 (1,147/2,077)**	**50 ** **(6 to 73)**	**16/42 ** **(1,147/2,077)**

CI: confidence interval; VE: vaccine effectiveness.

VE against any influenza and VE against influenza A were higher early in the season than late in the season (Breslow-Day, p < 0.05).

### Weekly changes in vaccine effectiveness

Crude VE against influenza A first became statistically significant in week 49, when it reached 69% (95% CI: 46 to 82) (Table 6). VE then gradually decreased from 60% (95% CI: 47 to 70) in week 51 of 2014 to 42% (95% CI: 34 to 50) in week 8 of 2015 and stabilised.

**Table 6:** Effectiveness of trivalent inactivated influenza vaccine against influenza A in children aged 6 months to 15 years, cumulative data, by week, influenza vaccine effectiveness study, Japan, November 2014 to March 2015 (n=3,752)

Year	Week	Type A positive		Influenza-negative		Vaccine effectiveness (95% CI)
		Vaccinated	Unvaccinated	Vaccinated	Unvaccinated	
2014	45	0	0	0	1	NA
	46	0	2	4	10	NA
	47	3	12	16	17	73 (-12 to 94)
	48	12	26	42	51	44 (-24 to 75)
	49	23	66	110	98	69 (46 to 82)
	50	50	121	182	162	63 (46 to 75)
	51	104	218	281	235	60 (47 to 70)
	52	199	358	381	327	52 (40 to 62)
2015	1	307	484	476	391	48 (37 to 57)
	2	368	560	554	459	46 (35 to 55)
	3	446	683	633	525	46 (36 to 54)
	4	515	780	710	579	46 (37 to 54)
	5	580	853	790	631	46 (37 to 53)
	6	623	898	849	688	44 (35 to 51)
	7	644	918	901	726	43 (35 to 51)
	8	656	924	949	769	42 (34 to 50)
	9	668	930	983	815	40 (32 to 48)
	10	674	939	1,031	844	41 (33 to 49)
	11	675	942	1,068	873	41 (33 to 49)
	12	676	950	1,112	900	42 (34 to 50)
	13	681	952	1,141	927	42 (34 to 49)
	14	681	952	1,147	930	42 (34 to 49)

CI: confidence interval; NA: not analysed; VE: vaccine effectiveness.

VE against influenza B, on the other hand, was rather unstable because of the small number of patients (data not shown).

### Number of doses of vaccine

Two doses of influenza vaccine did not provide better protection against influenza A in children of 6 months to 12 years of age than a single dose, even though two doses of trivalent IIV were recommended for that age range. The OR of two doses (cases/controls, 451/800) vs one dose (164/294) was 1.01 (95% CI: 0.81 to 1.26) for influenza A and 1.35 (95% CI: 0.37 to 4.86) for influenza B (crude data).

### Vaccine coverage

The proportion of vaccine coverage calculated for the IRDT-negative enrollees was 55% (1,147/2,077). By age group, it was: 6‒11 months, 22% (30/136); 1‒5 years, 61% (804/1,312); for 6‒12 years, 51% (264/519); and 13‒15 years, 45% (49/110).

## Discussion

Estimations of the effectiveness of influenza vaccine by a TNCC design have been reported annually in recent years [20-22], and the TNCC design has become the standard design for assessing VE. In this study, we used the results of IRDTs as a basis for estimating VE using the TNCC design in children who had received trivalent IIV during the 2014/15 season, since almost all children with a fever receive an IRDT during an influenza epidemic [23], resulting in a large enrolment for this study.

The overall adjusted VE for prevention of laboratory-confirmed medically attended influenza illness in this large study of 3,752 children was 38% (95% CI: 28 to 46). Most cases (97.5%) had been infected by influenza A virus, and VE was 37% (95% CI: 27 to 45) in the influenza A group. Because over 99% of the influenza A viruses detected in Japan in the 2014/15 season were A(H3N2) viruses, the results of our study demonstrated that trivalent IIV was effective against the drifted influenza A(H3N2) in children. VE against influenza B, on the other hand, was not statistically significant because there were only 42 influenza B patients.

The majority, 88.3%, of the haemagglutinin (HA) sequences of the influenza A(H3N2) viruses isolated during the 2014/15 season and analysed at the Yokohama City Institute of Public Health belonged to subclade 3C.2a of clade 3C.2, and the National Institute of Infectious Diseases has reported that subclade 3C.2a accounted for the major epidemic A(H3N2) viruses in Japan in the 2014/15 season [15]. Consequently there have been genetic and antigenic mismatches between most epidemic A(H3N2) strains in Japan and the vaccine strains that have been used, as has been reported in Canada [5], the UK [6], and the US [7]. The low VE in the 2014/15 season, when the dominant influenza virus was A(H3N2), was postulated to be attributable to mutations in the egg-adapted A(H3N2) vaccine strain [24] as well as to a mismatch due to antigenic drift of the virus.

According to the interim estimates of 2014/15 VE in Canada [5], little or no VE was observed, because the adjusted VE against influenza A(H3N2) for all ages was − 8% (95% CI: − 50 to 23). Based on the end-of-season VE results for 2014/15 in the UK [25], the adjusted VE for all ages against influenza A(H3N2) was 29.3% (95% CI: 8.6 to 45.3). It was 29.4% for those 18 years of age and over, which was attributable to the effect of the IIV alone, but for those aged under 18 years, it was only 19.1%, which was attributable to the combined effect of both the LAIV and IIV, and was not statistically significant. The end-of-season VE results for 2014/15 in the US [7] showed that the adjusted VE for all ages against influenza A(H3N2) was 13% (95% CI: 2 to 23). However, none of these recent reports [5,7,25] clearly demonstrated VE of IIV in children. The results of our study showed that trivalent IIV provided low but significant protection against influenza A(H3N2) virus infection in children in the 2014/15 season in Japan, despite marked antigenic drift in the epidemic virus. In a previous paper, we reported having found that trivalent IIV was highly effective in protecting against influenza A(H3N2) virus infection irrespective of whether there had been marked antigenic drift [3].

The widespread circulation of influenza A(H3N2) viruses in the 2014/15 season provided an opportunity to compare VE according to age group. Although significant protection against influenza A(H3N2) illness was demonstrated in the 1- to 12-year-old group, VE was not statistically significant in the 6- to 11-month-old group or 13- to 15-year-old group. Similarly low or no effectiveness was observed in both the 6- to 11-month-old group and 13- to 15-year-old group in our study of VE in the 2013/14 season [14].

The results of the present study showed that the influenza vaccine was not effective against influenza A (-5%, 95% CI: -139 to 54) in 6- to 11-month-old infants. Similarly, no significant VE was shown against influenza A in infants in the 2013/14 season (21%, 95% CI: -87 to 67) [14]. Our studies in these two consecutive seasons showed that trivalent IIV was not effective against influenza A(H1N1)pdm09 or A(H3N2) in infants. However, the number of infants enrolled was relatively small, and further studies are needed.

We unexpectedly found that VE was low in adolescents (the 13–15 years age group), in the two consecutive seasons 2013/14 and 2014/15. In the 2013/14 season, both influenza A(H3N2) and A(H1N1)pdm09 were circulating in Japan [26], and no statistically significant VE against influenza A was observed in the 13- to 15-year-old group [14]. VE against influenza B was not statistically significant either [14]. Although we cannot explain this low or absent VE in adolescents, similar results, including low VE of trivalent IIV against influenza A(H3N2) and B in adolescents, were reported during the 2012/13 season in the US [27].

A meta-analysis showed no convincing evidence that influenza vaccine reduces mortality, hospitalisations, or serious complications in children [28]. However, the results of our previous study demonstrated that influenza vaccination was highly effective in reducing hospitalisation of children infected with influenza A in the 2013/14 season. In the present study, which covered the period of the widespread epidemic caused by the drifted influenza A(H3N2), it reduced such admissions of children infected with influenza A by 55%. Although the criteria for hospitalisation vary from country to country, our studies conducted two years in row demonstrated VE in reducing hospitalisation for influenza A in children in Japan, where over 90% of the children with influenza-like illness (ILI) enrolled in the present study were brought to clinics within 48 hours after the onset of illness and 96% were treated with NAIs if their IRDT was positive. There are recent reports from other countries showing that influenza vaccination was associated with reduced hospitalisations [29] and reduced clinical severity in children [30].

Our previous study showed that VE against influenza A and B decreased by ca 10% in the latter half of the epidemic [14]. The present study showed that VE against influenza A declined greatly over the course of the epidemic, from 46% in November, December, and January to 13% in February and March. Thus, persistence of VE depends on the type and subtype of influenza viruses and the match between vaccine strain and epidemic virus.

The weekly changes in VE shown in this study demonstrated the major advantage of a TNCC design based on IRDT results. It is easy to calculate VE every week in Japan. VE against influenza A gradually declined every week from 69% in week 49 of 2014 to 42% in week 8 of 2015.

Two doses of influenza vaccine have been reported to be necessary to provide sufficient protection in children [4,31-33], and our previous study [14] showed that two doses were needed to optimise protection against influenza A in children. However, the results of the present study show that a single dose of influenza vaccine was as effective as two doses of vaccine in protecting against influenza A in children. The difference between the results in the two season can be explained by the fact that the epidemic in the 2014/15 season started and peaked much earlier than the 2013/14 epidemic [15] and even though many children received only one dose in the 2014/15 season, adequate VE was maintained. If the 2014/15 epidemic had started later, there might have been a difference in VE between two doses and one dose.

The limitations of this study need to be considered. Unlike most previous TNCC studies based on RT-PCR data, our study was based on the results of IRDTs. Although using IRDTs in TNCC studies has been reported to possibly result in underestimations of VE [34,35], Suzuki et al. found no difference between VE estimated on the basis of IRDT results and VE estimated on the basis of PCR data [36], and the VE results in our previous study were consistent with the results based on RT-PCR findings reported in another study [14]. VE estimates have been found to be much less influenced when the sensitivity of the diagnostic method used is over 80%, although low specificity has been found to cause greater bias in VE estimates [35]. The sensitivity of the IRDT kit used in this study (Espline Influenza A and B-N kit) is 85.1% to 92.4% for influenza A and 71.6% to 91.2% for influenza B, and its specificity is 97.6% to 100% [37]. Moreover, over 90% of the children with ILI were brought to our clinics within 48 hours of illness onset. By contrast, in most of the TNCC studies based on the RT-PCR tests, the patients were enrolled within 7 days after illness onset, suggesting that influenza virus could not have been detected even by the RT-PCR tests [38,39].

A TNCC design based on IRDT results is limited from an epidemiological standpoint, since the VE against each subtype of influenza A or especially against each lineage of influenza B cannot be determined. However, from a clinical standpoint, a TNCC design based on IRDT results has various advantages. VE can be communicated easily to the Japanese population during the very early stages of an influenza epidemic, and more importantly, VE against hospitalisation can be easily calculated.

In the near future, VE estimated by a TNCC assessment based on IRDT results will be reported weekly in many areas of Japan. The large number of patients in Japan who receive an IRDT makes it possible to estimate VE with considerable precision, and the most appropriate vaccination policy will be established based on the data obtained.
